# Gastric Decompression Decreases Postoperative Nausea and Vomiting in ENT Surgery

**DOI:** 10.1155/2014/275860

**Published:** 2014-04-02

**Authors:** Kerem Erkalp, Nuran Kalekoglu Erkalp, M. Salih Sevdi, A. Yasemin Korkut, Hacer Yeter, Sertuğ Sinan Ege, Aysin Alagol, Veysel Erden

**Affiliations:** ^1^Istanbul Bagcilar Educational and Training Hospital, 34200 Istanbul, Turkey; ^2^Ventigoo ENT and Balance Center, 34180 Istanbul, Turkey; ^3^Istanbul Sisli Etfal Training Hospital, 34360 Istanbul, Turkey; ^4^Istanbul Educational and Research Hospital, 34104 Istanbul, Turkey

## Abstract

There is a passive blood flow to the stomach during oral and nasal surgery. It may cause postoperative nausea and vomiting (PONV). We researched the relationship between gastric decompression (GD) and severity of PONV in ear, nose, and throat (ENT) surgery. 137 patients who have been into ENT surgery were included in the study. In Group I (*n* = 70), patients received GD after surgery before extubation; patients in Group II (*n* = 67) did not receive GD. In postoperative 2nd, 4th, 8th, and 12th hours, the number and ratio of patients demonstrating PONV were detected to be significantly more in Group II as compared to Group I. PONV was also significantly more severe in Group II as compared to Group I. In Group I, the PONV ratio in the 2nd hour was significantly more for those whose amounts of stomach content aspired were more than 10 mL as compared to those whose stomach content aspired was less than 10 mL. In the 4th, 8th, and 24th hours, there is no statistically significant difference between the stomach content aspired and PONV ratio. GD reduces the incidence and severity of PONV in ENT surgery.

## 1. Introduction

Postoperative nausea and vomiting (PONV) may develop due to risk factors associated with surgery as well as characteristics of patients [1]. General incidence of PONV is in the range of 20–30% while it increases up to 30–70% after ear, nose, and throat (ENT) surgery [2–4]. Risk factors may include early age, female sex, tobacco addiction, type of the surgery applied, history of PONV after surgical procedures previously applied, motion sickness, gastroparesis, obesity, and postoperative analgesic use of opioids [2, 3, 5].

PONV management includes single dose or multimodal antiemetic use, steroid treatment, and intravenous fluid support [6]. Additionally, it is suggested to reduce the use of opioid analgesics in postoperative pain control and it is preferred to use nonopioid analgesics [6]. Other treatment options are P-6 acupuncture point stimulation and perioperative gastric decompression (GD) practices [7, 8].

In ENT surgery, there is a passive blood flow to the stomach during the process [9, 10]. In literature, some studies demonstrate that removing swallowed blood through GD decreases PONV incidence, while there are also reports stating that it increases the incidence [9, 10].

In our study, we have researched the relationship between GD and PONV incidence and the correlation of the amount of contents aspired from the stomach with the severity of PONV.

## 2. Material and Method

Our study was conducted on 137 patients undergoing tonsillectomy, septoplasty, tympanoplasty, tympanomastoidectomy, adenoidectomy, adenotonsillectomy, microlaryngeal surgery, septorhinoplasty, dacryocystorhinostomy, functional endoscopic sinus surgery, neck dissection, thyroidectomy, septal concha radiofrequency application, and uvulopalatoplasty in an ENT surgery room, after obtaining an approval certificate from the hospital's local ethics committee and individual permissions from patients. In a six-month period (September 2010–April 2011), patients aged between 18 and 65 years and whose ASA risk condition is I/II/III were included in the study.

Patients with previous PONV history, motion sickness history, antiemetic drug allergy history, Meniere's disease, major cancer surgery, a hunger period shorter than 8 hours before elective surgery, kidney and liver disease, upper respiratory system pathology, a history of using antiemetic drugs, morbid obesity, and pregnancy were excluded from the study. Additionally, patients were excluded from the study in cases where major complications developed during surgery; surgery lasted for longer than 180 minutes and antiemetic drugs or steroids were used in the perioperative period.

All the cases were examined in terms of anesthesia one day before the operation. With the laboratory tests, all patients were controlled in terms of their blood count, coagulation parameters, electrolyte values, liver enzyme values (SGOT, SGPT), BUN, creatinine, and hunger blood glucose values.

The patients were separated into two groups by a nurse in the preparation room through a closed envelop method. While patients in Group I (*n* = 70) which is determined as study group received GD, the patients in Group II (*n* = 67) which is determined as control group did not receive GD.

Patients were taken to the operating room without receiving premedication. After tree-channel electrocardiogram (ECG), noninvasive blood pressure and peripheral oxygen saturation (SpO_2_) monitoring were provided, the vascular access was opened with 22 gauge (G) intravenous cannula from the veins on the left hand, and Isolyte solution was infused with the pace of 8 mL/kg/hour. In anesthesia induction, 2-3 mg/kg propofol, 1 *μ*g/kg fentanyl, and 0.6 mg/kg rocuronium for neuromuscular blockage were applied. After applying 6 L-100% oxygen for 3 minutes through the face mask, endotracheal intubation was performed. Ventilation was provided for all patients in volume-controlled ventilation mode as 50-50% air-O_2_, 2 L/min. flow, and 1-2% sevoflurane (Datex-Ohmeda, S/5 Avance, GE Healthcare, USA). During ventilation, respiration frequency was adjusted to 12/min, inspiration and expiration rate was adjusted to be 1 : 2, and tidal volume was adjusted to be 8 mL kg^−1^. The patients received 20 mg/kg iv paracetamol as postoperative analgesic. After the surgery finished, inhalation agents were closed and 100% oxygen was applied as 6 L/dk. The patients in Group I received CH14, 53 cm Mully suction catheter (Unomedical, ConvaTec Limited, UK), and GD process via oral access through airway (number 3). After the distal of the suction catheter was placed into the stomach, we waited for passive drainage of air. Then, the stomach contents were aspired with a 50 mL feeding injector. Calculating the intraluminal volume of CH14, 53 cm suction catheter as 5 mL, the amount of stomach content suctioned with an injector was measured. After the drainage of stomach content through suction was finalized, the catheter was removed. The amount of stomach content aspired during the period of GD process (minute) was recorded as “less than 10 mL” or “more than 10 mL.” Oral airway (number 3) was also placed to the patients in Group II but GD was not applied. After the spontaneous respiration began in patients, neuromuscular block, atropine as 0.01 mg/kg, and neostigmine as 0.05 mg/kg were recovered. When the spontaneous respiratory effort was enough, the patients were extubated. After extubation, patients were kept in a 30° head-up position and all patients received 6 L/min oxygen. PONV was evaluated as being present or absent as compared to the severity in the 1st, 4th, 8th, and 24th hours. In patients who demonstrated PONV, it was recorded as mild (mild nausea, vomiting once, and nausea through an outer stimulant [eating, drinking, and motion]), moderate (vomiting twice, mild nausea without an outer stimulant, and antiemetic medication need once), and severe (vomiting more than twice, severe nausea, antiemetic medication need more than once) [11]. Patients with moderate and severe PONV received 10 mg metoclopramide iv in antiemetic medication. Neither of the patient groups received opioid or antiemetic medication in intraoperative or postoperative periods.

*Statistical Method.* In supplementary statistics of the data, mean, standard deviation, ratio, and frequency values were used. The distribution of the variants was controlled through a Kolmogorov-Smirnov test. In analysis of quantitative data, an independent sampling *t*-test and Mann-Whitney *U* test were used. In analysis of the qualitative data, a chi-square test was used; when the conditions could not be obtained for a chi-square test, a Fischer test was used. *P* < 0.05 was considered to be statistically significant. The SPSS 20.0 program was used for analysis.

## 3. Results

Demographic data and anesthesia and surgery duration of patients in Group I and Group II were demonstrated in Table 1.

Average GD duration in Group I was 127.88 ± 45.54 seconds.

In postoperative 2nd, 4th, 8th, and 24th hours, the number and ratio of patients demonstrating PONV were detected to be significantly more in Group II as compared to Group I (Table 2). PONV was also significantly more severe in Group II as compared to Group I (Table 3).

In Group I, the PONV ratio in the 2nd hour was significantly more for those whose amounts of stomach content aspired was more than 10 mL as compared to those whose stomach content aspired was less than 10 mL. In the 4th, 8th, and 24th hours, there is no statistically significant difference between the stomach content aspired and PONV ratio (Table 4).

## 4. Discussion

PONV incidence may increase up to 70% in patients undergoing ENT surgery [1, 4]. In these patients, for whom inpatient surgery is quite common, PONV is an important indicator of patient satisfaction [12]. In addition to psychological effects, PONV may lead to airway obstruction, aspiration pneumonia, subcutaneous emphysema, bleeding, opening and latency of healing in incisions, increase of intracranial pressure, dehydration, electrolyte imbalance, malnutrition due to insufficiency of oral intake, lengthening in hospitalization period, and increased costs [1, 13, 14].

In patients undergoing ENT surgery, it is reported that the reason of PONV is blood flow to the stomach during the intraoperative and postoperative periods and surgical procedures applied in intraoperative period [6, 8, 15]. Direct stimulation of the chemoreceptor trigger zone through mucosal damage and accompanying pharyngeal edema is also effective in PONV formation [6, 8, 15]. Another reason for PONV is that oropharynx and stomach chemoreceptors and mechanoreceptors are activated when the trigeminal nerve is stimulated [6, 8, 15, 16]. The factors increasing PONV risk may include postoperative pain, anxiety, vertigo, early mobilization, early oral intake, and opioid analgesics [17, 18]. Additionally, it has been stated that PONV risk associated with gastric distension may increase in patients where the air pressure increases over 25 cm H_2_O during ventilation with a mask [15].

Many methods have been used in order to decrease PONV incidence in ENT surgery. Prophylactic and treatment-purpose use of antiemetics is the most common [6, 18, 19]; it is frequently applied by anesthesiologists in gastric decompression. We are of the opinion that every anesthesiologist should apply GD to patients whether the patient is aware or not. The orogastric method is more easily, safely, and frequently used than the nasogastric method [8]. For this reason, we applied gastric decompression through the orogastric method in our study. We did not encounter any complications during or after application.

In literature, the number of studies searching for the effect of GD application in ENT surgery on PONV incidence is very low. Pasternak [20] reported that PONV and aspiration pneumonia risks can be eliminated by means of placing a gastric tube. Ferrari and Donlon [4] used prophylactic antiemetics in young patients undergoing tonsillectomy and applied GD in their studies and stated that the PONV incidence was 47% in the group where they used metoclopramide and 70% in the group where they did not use it. The PONV ratio in patients whom Ferrari applied prophylactic antiemetic together with GD is almost twice as much as the PONV ratio (21%) in patients to whom we applied only GD in this study. We can relate the low PONV rate to the patient group who underwent various ENT surgeries in our study. Trepanier and Isabel [15] reported the PONV ratio in 265 patients undergoing surgery in different branches as 55% in the group receiving GD and 48% in the control group and argued that GD has no effect on PONV incidence [15]. However, in this study, it was stated that 35 patients had previous postoperative PONV histories and 21 patients had vertigo histories; these patients were not excluded from the study [15]. It was reported that in addition to the applied surgery type, use of opioids and nitrous oxide in anesthesia method also increases PONV incidence [3, 5, 15, 17]. PONV incidence is very variable in different ENT surgery types. For example, a 62–80% incidence of PONV following middle ear surgery has been reported [21]. The incidence of PONV was higher after middle ear surgery than tonsillectomy and the others [22, 23]. Our samples had been small heterogeneous surgery type about PONV incidence. Unfortunately it is the most limitation of recent study. Jones et al. [8] reported 85% PONV incidence in the group receiving GD and 74% PONV incidence in the control group in a study they conducted on 74 patients receiving adenotonsillectomy where they used 70% nitrous oxide and intravenous morphine sulfate through inhalation without using prophylactic antiemetic and steroid; they argued that GD application does not affect PONV incidence [8]. It was also stated that there is no correlation between gastric evacuation and PONV. Hovorka et al. [10] argued that GD application may be effective in reducing the frequency of PONV but cannot affect its severity. Burlacu et al. [2] reported that the need for postoperative antiemetic was 38.5% in the group receiving GD and 28.5% in the patients who did not receive GD, in 107 patients undergoing coronary artery bypass surgery using fentanyl and morphine sulfate. They argued that GD application does not have an effect on PONV incidence.

It is observed that the number of studies on this subject is low and there is no clear result on the effectiveness of GD. In our study, the PONV ratio was lower in the 4th and 24th hours in patients receiving GD. PONV incidence was lower, especially in the first 2 hours, in patients for whom the amount of content aspired from the stomach was lower than 10 mL as compared to those for whom the amount of content aspired from the stomach was more than 10 mL; namely, less stomach content means less PONV.

As a result, we can say that, in patients undergoing ENT surgery for whom we minimized PONV factors, GD applied just before extubation after the surgery reduces the incidence and severity of PONV. However, it should be noted that the more stomach content we aspire, the more frequently and severely PONV occurs. The number of studies pertaining to GD use should be increased in different surgeries, in different patient groups, and in different times and even in cases where risk factors are present. Since it is cheap and easily applicable, does not require special skills, and has a low complication rate, it can be preferred in adult ENT patients as an alternative for pharmacological treatment methods used today.

## Figures and Tables

**Table 1:** Demographic data and anesthesia and surgery duration of patients in Group I and Group II.

	Group I		Group II		*P*
	Mean ± SD/*n* %		Mean ± SD/*n* %		
Age (year)	33.5 ± 14.0		34.2 ± 13.0		0.245

Gender					
Female	29	41.4%	31	46.3%	0.568
Male	41	58.6%	36	53.7%	

BMI (kg/m^2^)	24.6 ± 4.2		23.6 ± 3.3		0.121

ASA					
I	58	82.9%	50	74.6%	0.238
II	10	14.3%	16	23.9%	
III	2	2.9%	1	1.5%	

Anesthesia duration (minute)	65.3 ± 38.6		71.2 ± 36.2		0.113

Surgery duration (minute)	52.4 ± 32.0		57.5 ± 35.0		0.065

Surgery type	Tonsillectomy *n* = 5 Septoplasty *n* = 9 Tympanoplasty *n* = 3 Tympanomastoidectomy *n* = 2 Adenoidectomy *n* = 6 Adenotonsillectomy *n* = 5 Microlaryngeal surgery *n* = 4 Septorhinoplasty *n* = 6 Dacryocystorhinostomy *n* = 3 Functional endoscopic sinus surgery *n* = 7 Neck dissection *n* = 4 Thyroidectomy *n* = 3 Septal concha radiofrequency application *n* = 9 Uvulopalatoplasty *n* = 4 (*n* = 70)		Tonsillectomy *n* = 4 Septoplasty *n* = 11 Tympanoplasty *n* = 2 Tympanomastoidectomy *n* = 2 Adenoidectomy *n* = 5 Adenotonsillectomy *n* = 6 Microlaryngeal surgery *n* = 5 Septorhinoplasty *n* = 7 Dacryocystorhinostomy *n* = 2 Functional endoscopic sinus surgery *n* = 5 Neck dissection *n* = 4 Thyroidectomy *n* = 4 Septal concha radiofrequency application *n* = 7 Uvulopalatoplasty *n* = 3 (*n* = 67)		0.216

**Table 2:** The number and ratio of patients demonstrating PONV in postoperative 2nd, 4th, 8th, and 24th hours,.

		Group I		Group II		*P*
		*n*	%	*n*	%	
PONV	First hour					
	None	63	90.0%	32	47.8%	
	PONV					
	Mild	3	4.3%	22	32.8%	0.000*
	Moderate	4	5.7%	8	11.9%	
	Severe	0	0.0%	5	7.5%	
	Fourth hour					
	None	57	81.4%	24	35.8%	
	PONV					
	Mild	9	12.9%	25	37.3%	0.000*
	Moderate	3	4.3%	14	20.9%	
	Severe	1	1.4%	4	6.0%	
	Eighth hour					
	None	62	88.6%	24	35.8%	
	PONV					
	Mild	7	10.0%	35	52.2%	0.000*
	Moderate	1	1.4%	6	9.0%	
	Severe	0	0.0%	2	3.0%	
	24th hour					
	None	67	95.7%	50	74.6%	
	PONV					
	Mild	3	4.3%	16	23.9%	0.000*
	Moderate	0	0.0%	1	1.5%	
	Severe	0	0.0%	0	0.0%	

**Table 3:** Severity of the PONV in Groups I and II.

	Group I		Group II		*P*
	*n*	%	*n*	%	
PONV					
First hour	7	10.0%	35	52.2%	0.000*
Second hour	13	18.6%	22	64.2%	0.000*
Eighth hour	8	11.4%	8	64.2%	0.000*
24th hour	3	4.3%	5	25.4%	0.000*

**Table 4:** The amount of stomach content aspired during the period of GD process (minute) was recorded as “less than 10 mL” or “more than 10 mL.” In Group I, the PONV ratio in the 2nd hour was significantly more for those whose amounts of stomach content aspired were more than 10 mL as compared to those whose stomach content aspired was less than 10 mL. In the 4th, 8th, and 24th hours, there is no statistically significant difference between the stomach content aspired and PONV ratio.

	The amount of stomach content				*P*
	Less than 10 mL		More than 10 mL		
	*n*	%	*n*	%	
PONV					
First hour	2	4.3%	5	21.7%	0.022*
Second hour	10	21.3%	3	13.0%	0.405
Eighth hour	6	12.8%	2	8.7%	0.615
24th hour	3	6.4%	0	0.0%	0.546
